# Towards plasma jet controlled charging of a dielectric target at grounded, biased, and floating potential

**DOI:** 10.1038/s41598-022-05075-4

**Published:** 2022-01-21

**Authors:** Elmar Slikboer, Olivier Guaitella, Enrique Garcia-Caurel, Ana Sobota

**Affiliations:** 1 LPP, CNRS, École Polytechnique, Sorbonne Université, Université Paris-Saclay, IP-Paris, 91128 Palaiseau, France; 2 Department of Applied Physics, EPG, Eindhoven University of Technology, Eindhoven, The Netherlands; 3 Laboratoire de Physique des Interfaces et des Couches Minces (LPICM), CNRS, École Polytechnique, Institut Polytechnique de Paris, 91120 Palaiseau, France; 4 Present Address: Department of Electrical Engineering and Electronics, Centre for Plasma Microbiology, University of Liverpool, Liverpool, UK

**Keywords:** Plasma physics, Imaging techniques, Imaging and sensing

## Abstract

Electric field and surface charge measurements are presented to understand the dynamics in the plasma–surface interaction of a plasma jet and a dielectric surface. The ITO coated backside of the dielectric allowed to impose a DC bias and thus compare the influence of a grounded, biased and floating potential. When imposing a controlled potential at the back of the target, the periodical charging is directly dependent on the pulse length, irrespective of that control potential. This is because the plasma plume is sustained throughout the pulse. When uncontrolled and thus with a floating potential surface, charge accumulation and potential build-up prevents a sustained plasma plume. An imposed DC bias also leads to a continuous surface charge to be present accumulated on the plasma side to counteract the bias. This can lead to much higher electric fields (55 kV/cm) and surface charge (200 nC/cm$^2$) than observed previously. When the plasma jet is turned off, the continuous surface charge decreased to half its value in 25 ms. These results have implications for surface treatment applications.

## Introduction

Cold atmospheric pressure plasmas are used for many applications, ranging from bio-medical^1,2^ to surface functionalization^3^, thanks to the non-equilibrium nature of the discharges leading to physicochemical processes at the plasma–surface interface. These include a plethora of reactive oxygen and nitrogen species (when air is present), (UV-)radiation, electric fields, and charge deposition^4^. A plasma jet device is often used to generate the cold discharges, where the electrode configuration and helium feeding gas allow for an open plasma plume to form where a target can be placed^5^.

The interactions between a plasma jet and a target are diverse and are the result of kHz-applied high voltage pulses. These cause ionization waves (also called plasma bullets) to travel from the inner electrode of the plasma jet, into the plasma plume onto the targeted material. Naturally, the properties of the targeted material play a significant role in the interaction^6–12^. A way to investigate the plasma–surface interaction is by using electro-optic targets and exploiting the Pockels effect^13–15^. This can be done with a Senarmont setup or a more advanced Mueller polarimeter as is used throughout this work. The Pockels effect describes the change in refractive index of the material as a function of externally induced electric fields. When the material is in direct contact with a plasma, the electric fields are induced by surface charge deposition and therefore this approach gives a way to examine the influence of the plasma on the target. This is complementary to other diagnostic techniques for the study of electrical properties of a plasma which focus on the characterization of the plasma plume, such as Thomson scattering, Stark broadening, Stark shift, or coherent anti-Stokes Raman scattering^11,16–20^.

The target’s dielectric constant (permittivity) is an important parameter that influences the perturbance the target has on the plasma jet operation and the amount of charge deposition that occurs. The electro-optic target used throughout this work has a dielectric constant of 56, therefore the plasma–target interaction examined throughout this work relates to relatively high permittivity targets. In^21^ the influence of the dielectric constant was examined and shown that when the permittivity is higher than 4 the plasma–surface interaction is comparable in terms of charge deposition and experienced electric field.

In this work the targeted material has an ITO coating on the backside, i.e. the side not exposed to the plasma jet. This allows to impose a DC bias and compare the effect of grounded, biased or floating potential on the dynamics of the plasma–surface interaction. The results of this work will be divided in two parts. The first will focus on the periodical charging during each high voltage pulse $\sigma _p$ and part two focuses on the accumulation of surface charge as a result of the DC bias creating a continuous charging $\sigma _c$.

## Results

### Periodical charging $\sigma _p$

**Figure 1:**
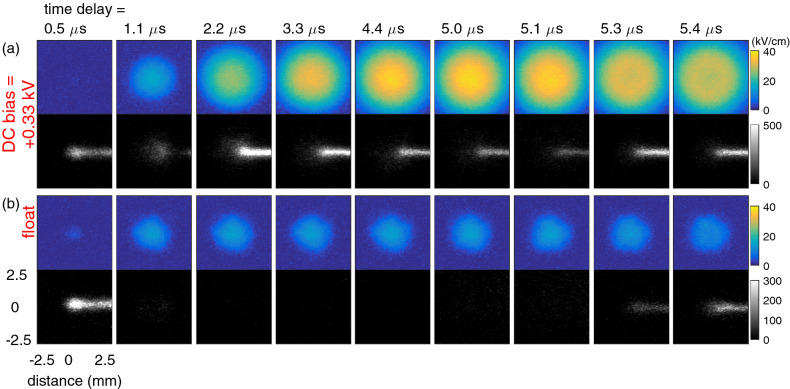
Induced electric field patterns (top row, kV/cm) together with light emission (2nd row, arb. units) from the plasma jet impacting at normal incidence seen at a 45 degree angle on the BSO surface for two cases: (**a**) a DC bias of + 0.33 kV and (**b**) floating potential. The impact point was at the center of the $5 \times 5$ mm$^2$ field of view and the plasma jet was located at the right hand side. The plasma was generated using 5 $\upmu$s pulses of 4 kV with a frequency of 5 kHz. A background image has been subtracted to focus on the dynamics during the impact.

**Figure 2:**
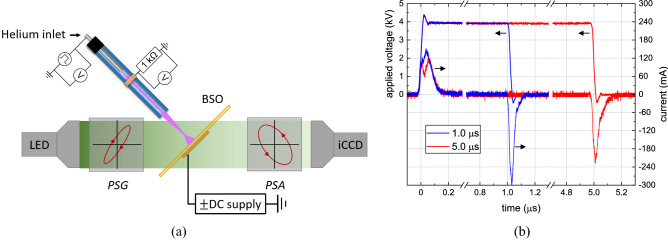
(**a**) The experimental setup used throughout this work, showing the BSO crystal and plasma jet in the center of the Mueller polarimeter in between the *polarizer state analyzer* (PSA) and *polarizer state generator* (PSG). On the backside of the BSO was an ITO layer connected to a DC power supply to impose a DC biased potential. (**b**) the voltage and current waveform for 1.0 and 5.0 $\upmu$s applied voltage pulses.

Figure 1 shows the obtained electric field patterns induced by a plasma–surface interaction using 4 kV pulses of 5 $\upmu$s. The top row shows the electric field and light emission when a DC bias of + 0.33 kV was applied. The results shown in the bottom row were obtained when the ITO coating was at floating potential since no DC bias potential was applied. Measurements were taken at various points throughout the applied HV pulse (see Fig. 2b) by varying the time delay.

Measurements timed at a 100 $\upmu$s after the pulse had finished were used to subtract any background and focus on the periodically induced electric field and surface charge density $\sigma _p$ during each applied pulse. The surface charges that have been accumulated in time as a result of the DC bias and formed a continuous surface charging $\sigma _c$ were included in the background and therefore subtracted. They will be discussed in part 2 of this work.

The images were obtained using an exposure time of the iCCD camera of 100 ns and accumulating 60 exposures. The light emission images were needed to subtract as *dark images* from the Mueller matrices to analyze and obtain the induced electric field. Therefore the light emission was seen as shown in Fig. 2a at a 45 degree observation angle. The horizontal axis has been multiplied by $\sqrt{2}$ to account for this projection. The time delay was varied with respect to the start of the 5 $\upmu$s pulses. It can be seen for both cases that the initial contact between the plasma and the surface was made after 500 ns. The impact point at the surface is in the center of the $5 \times 5$ mm$^2$ field of view. No difference was found for the impact time at the surface depending on the DC bias, but this is possibly due to the time resolution of a 100 ns. The plasma jet was oriented on the right, meaning the plasma plume was visible on the right-hand side of the impact point.

Charges were deposited after the initial contact of the plasma with the surface. The resulting electric field is clearly visible at a delay of 1.1 $\upmu$s and the difference between both cases were small. However, in the case where a DC bias was applied it is observed that after the light emission first decreased at 1.1 $\upmu$s there is an increase and continued plasma light emission observed from 2.2 $\upmu$s. This light emission was not observed when the surface was at floating potential. As a result of this second phase in the plasma dynamics, the induced electric field profile continued to expand and increase when a DC bias was applied. The total spread at the surface appears bigger than the light emission at the impact point. However it is still possible there is plasma propagation over the target surface but with a significantly decreased light emission compared to the light emission from the plasma plume area. The electric field did not increase further when there was no DC bias.

The maximum electric field induced by the plasma surface interaction was 38 kV/cm for the DC biased case and 18 kV/cm for the floating potential case. The electric field decreased after the 5 $\upmu$s pulse had finished and an increase of light emission was observed at 5.3 and 5.4 $\upmu$s.

**Figure 3:**
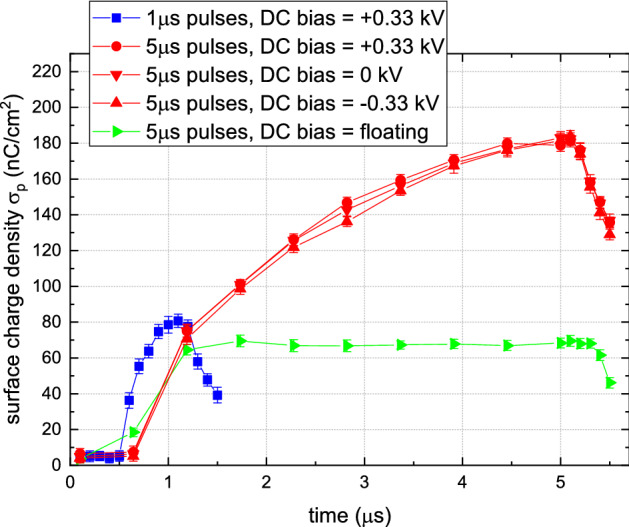
Surface charge density $\sigma _p$in the center of the impact point as a function of time for various cases of DC bias and pulse width. Similar as Fig. 1, measurements were done using 100 ns exposures and a background image was subtracted to focus on the repetitive dynamics during the impact.

This can be seen as well in Fig. 3 where the electric field in the center of the impact point has been converted to surface charge using Eq. (2). Additional to the measurements shown in Fig. 1, also the case of DC bias at $-\, 0.33$ kV and 0 kV have been added together with the results when 1 $\upmu$s pulses were applied with a DC bias of $+ \,0.33$ kV.

There was no significant difference between the cases of a DC bias of $\pm 0.33$ kV or 0 kV. Surface charge density $\sigma _p$ continued to increase throughout the pulse up to 180 nC/cm$^2$. For the floating case it was clearly seen that the surface charge remained constant at 70 nC/cm$^2$. When 1 $\upmu$s pulses were applied the surface charge reached a value of 80 nC/cm$^2$ at the end of the pulse, which matched well the value reached at that time instance when 5 $\upmu$s pulses were applied. This suggests that the initial charging during each pulse is similar, while the second phase of the dynamics with the continued plasma interaction only becomes significant after 1 $\upmu$s as is visible in Fig. 1. Both the induced electric field and $\sigma _p$ are positive, independently of the polarity of the applied bias potential. This is a result of the positive applied pulses which imposes the polarity of the discharge front reaching the surface^15,22^, and $\sigma _c$ which is present as well and will be discussed next.

### Continuous surface charge $\sigma _c$

As stated in the previous section, a background was subtracted to focus on the repetitive charging that occurs each HV period by the deposition and removal of charge during the plasma–surface interaction. This background was taken at a time delay of 100 $\upmu$s after the pulse ended. When no DC bias was applied, a measurement at this time delay showed that all the charge deposited during the HV pulse (i.e. $\sigma _p$) had been removed by then. Therefore when a DC bias was applied, any charge detected at this time delay is defined as the continuous surface charge $\sigma _c$. This background also includes birefringence induced not by electric field but by internal strain in the BSO material caused by temperature gradients^23,24^. This background is now analyzed further.

**Figure 4:**
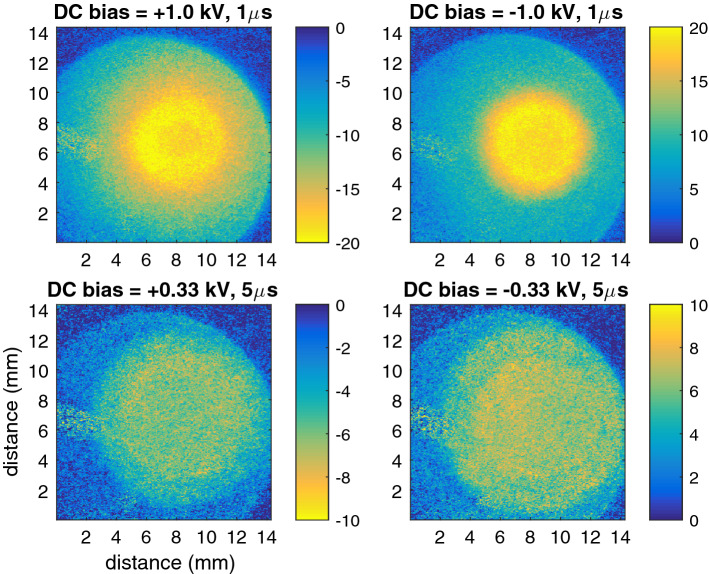
Electric field profiles (kV/cm) induced by the continuous surface charge pattern $\sigma _c$ in various cases of DC bias (polarity and amplitude) and pulse width. They have been obtained from the background measurements used to make Figs. 1 and 3 performed at a timing delay of 100 $\upmu$s after each applied HV.

The constant surface charge is a result of the applied DC bias. This means that when the ITO layer is grounded (zero bias), there is no continuous surface charge only the periodical surface charging as shown in the first section. Figures 1 and 3 indicate the dynamics of the periodical plasma–surface interaction is unaffected by the polarity or magnitude of the applied DC-bias. This suggests that the heating, and thus temperature gradients, of the target is similar in all cases. This allows for the removal of the internal strain induced birefringence in all cases by subtracting the birefringence obtained when the ITO was grounded. This leaves the constant surface charge patterns as shown in Fig. 4.

A larger field-of-view is shown than in Fig. 1. This reveals the circular ITO coating since everywhere where the coating was not present the electric field is closest to zero. The semi-spherical golden connectors to the ITO layer partially blocked the field of view and can be seen on the left-hand side of the images. Both the profile and the amplitude of the obtained electric field depended on the DC bias (polarity and amplitude) and the pulse width of the applied HV pulses.

The amplitude and polarity of the obtained electric field relate to the amplitude and polarity of the applied DC bias. A value of ± 20 kV/cm was observed when a DC bias of ± 1 kV was applied while a value of ± 7 kV/cm was observed for a DC bias of ± 0.33 kV. This matched the expected value of the applied bias potential divided by the thickness of the sample of 0.5 mm.

**Figure 5:**
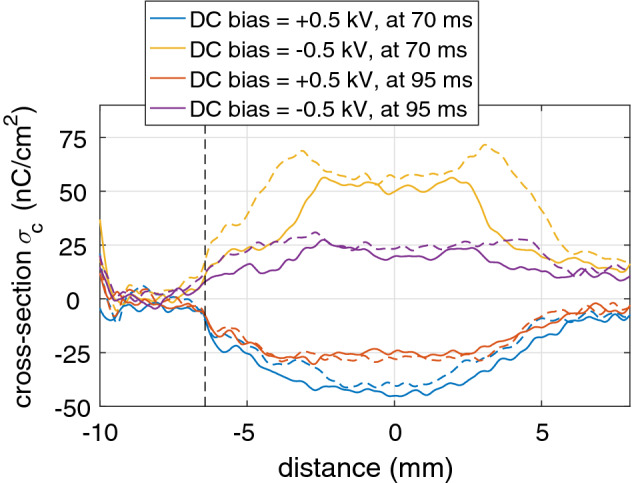
Continuous surface charge densities $\sigma _c$ for $\pm\, 0.5$ kV DC bias along a vertical cross-section going through the impact point. The plasma jet was operated in burst mode (70 ms on, 30 ms off) and solid lines show the results during plasma-off time obtained for 4kV pulses of 1 $\upmu$s and dashed lines for 5kV pulses. The border of the ITO layer is show at − 6.5 mm.

The shape of the profile was different depending on the polarity of the applied DC bias. To examine the dynamics and decay of the continuous charge profile, the plasma-jet was operated next in burst mode where it was repeatedly turned on for 70 ms (at the same settings as in the previous section) and then turned off for 30 ms. Figure 5 shows the decay of the continuous charge profile during the plasma-off time when the plasma jet was operated in burst mode.

The continuous charge profile of the electric field was used to calculate the surface charge density using Eq. (2) and a vertical cross-section through the impact point was plotted in Fig. 5. Results are shown for 1 $\upmu$s pulses of 4 kV in solid lines and for 1 $\upmu$s pulses of 5 kV in dashed lines. Lines in yellow and blue show the charging profile right after the train of pulses of 70 ms had finished, while lines in purple and red show how much the profile has changed 25 ms after the plasma jet had been turned off. The DC bias of ± 0.5 kV was still active. This caused a surface charge $\sigma _c$ value of approximately ± 50 nC/cm$^2$ to be present, which decayed to approximately ± 25 nC/cm$^2$ at 95 ms.

A striking difference was observed in the profile of the surface charge (and electric field) caused by the polarity of the applied DC bias. When a negative bias was applied (and thus a positive surface charge and electric field is observed) the profile was flat in the center and convex on the outer edges. For a positive bias a wider profile was observed due to the more concave profile of charge deposition.

The continuous charge profile did not change significantly when 5 kV pulses were applied to generate the plasma instead of 4 kV. Just for negative applied DC biases it appeared that the continuous surface charge had become wider and the value at the edges had surpassed the expected value for DC bias potential divided by the thickness. The profile for positive applied DC bias potentials seemed to be unaffected.

## Discussion

Mueller polarimetry has been applied to examine the charging of a dielectric surface under the exposure of a $\upmu$s-pulsed plasma jet operated at 5 kHz. The ITO coating on the backside of the dielectric sample was used to impose and examine the effect of a DC biased potential for the operation of the plasma jet and deposition of surface charges and resulting electric field.

The electric field values reported in this work (up to 35 kV/cm) are much larger than the reported values of electric field due to surface charge of 3–6 kV/cm in previous works^14,25^. However, caution has to be taken because the electric field was automatically twice higher because surface charge were present on the front and back of the dielectric sample due to the ITO coating with a DC bias, hence Eq. (2) was used. Nonetheless, significantly more charges were deposited, up to 80 nC/cm$^2$ for 1 $\upmu$s pulses and 180 nC/cm$^2$ for 5 $\upmu$s pulses. The increased amount of charge deposition is a direct consequence of the DC bias.

Previous research^15^ has shown surface charge densities on a dielectric target for a helium AC jet of approximately 22 nC/cm$^2$ and comparison with simulation^26^ for a 1 $\upmu$s pulsed helium jet showed densities of 35 nC/cm$^2$. A different type of a helium AC plasma jet^27^ showed charge deposition of 6 nC/cm$^2$. This indicates the wide range of charge deposition that occurs during the plasma–surface interaction imposed by an atmospheric pressure plasma jet. In order to have control over the plasma–surface interaction the dependencies with other operating parameters have to be understood.

The influence of the grounding conditions of a target under plasma exposure has been examined before regarding the electronic properties of the discharge. For a different coaxial-plasma jet operated using argon gas instead of helium and 5 $\upmu$s pulses (16 kHz), it was shown that the resistance of a conductive liquid target towards the ground determines whether it behaves as a DBD-type discharge (for a high impedance path to ground) or a DC-type (low impedance path to ground)^20,28^. This had significant consequences for the temporal dynamics of electron density and temperature in the center of the plasma plume.

The measurements done in this work focused on the periodical charging and the continuous surface charge. The effects the DC bias (amplitude and polarity) and HV pulses (amplitude and pulse width) have on these two types of charging are summarized for each point:

- *DC bias amplitude* The periodical charging $\sigma _p$ during the applied HV pulses does not seem to depend significantly on the amplitude of the applied DC bias. This is a result of the continuous surface charge $\sigma _c$ that accumulated in time, which does significantly depend on the amplitude. The continuous surface charge matches the imposed potential and therefore masks the DC bias. This limits the influence it has on the pulse-to-pulse impact and periodical charging $\sigma _p$ which is always positive. However because a DC bias is present the HV pulse duration does become more important as will be explained. The continuous charge density $\sigma _c$ in the center of the impact point relates to the expected electric field of the bias potential divided by the thickness of the dielectric target.
- *DC bias polarity* The polarity of the applied DC bias potential changes the electric field’s polarity inside the dielectric target of the continuous surface charge $\sigma _c$. This is at least partly because charges generated by the plasma jet of opposite polarity are accumulated at the surface. Due to the nature of the charges (e.g. electrons or negative ions versus positive ions) the spatial profile of the continuous charge depends on the DC bias polarity as well. A wider spread is observed when a positive bias is applied and thus negative charges are accumulated by the plasma at the surface. This is due to the larger mobility of negative charges on the surface and is a known feature for a streamer’s footprint. This has been shown e.g. experimentally^29,30^ or in simulation^31^.
- *HV amplitude* the amplitude of the $\upmu$s HV pulses applied to operate the plasma jet influence the periodical surface charge deposition and electric field during impact. This is at least partly because the HV amplitude significantly alters the velocity of the ionization waves travelling from the plasma jet to the dielectric surface. This is a normal feature for streamer propagation^32^. As a result this changes the impact time and thus the charging time till the end of the pulse^26^. With a higher applied voltage the impact occurs earlier within the applied pulse and more charges can be accumulated at the surface inducing a higher electric field. The spreading on the surface increased as well for higher applied voltages^33^. This influences the continuous surface charge when a negative DC bias is applied.
- *HV pulse duration* the length of the HV pulses is a direct tool to control the amount of charging during the HV pulse when a DC bias potential is applied. As stated this is independent of the polarity or amplitude of the bias. When the DC bias is not present and the dielectric sample is at floating potential the influence of the pulse duration is more limited. This is because the discharge from the plasma jet is not sustained throughout the entire pulse duration. Charges deposited at the surface of the dielectric cause the floating potential to rise, which decreases the electric field in the gap between the capillary and the sample (i.e. where the plasma plume is). When the electric field becomes too low the light emissions drop and the charge deposition at the surface is halted. When a DC bias is imposed this does not happen since charges deposited at the surface by the plasma cause other charges to be moving towards (or away from) the ITO coating. This counteracts the possible potential buildup and therefore the electric field in the gap will not be reduced and the plasma is sustained throughout the duration of the HV pulse. This causes charge deposition to continue. It also means charges are able to spread further along the surface, which influences the profile of the continuous surface charge.

## Methods

### Experimental setup

The experimental setup used for this work is shown in Fig. 2a similar as used in Ref.^26^. The 0.5 mm thick electro-optic BSO crystal (Bi$_{12}$SiO$_{20}$) was examined with the Mueller polarimeter at a 45 degree angle and placed in between the *polarizer state analyzer* (PSA) and *polarizer state generator* (PSG). A Mueller polarimetry setup is a more advanced diagnostic to study eletro-optic targets like BSO which can also be studied with a more simplified Sénarmont setup^14,15,34^. The atmospheric pressure plasma jet impacted the BSO at normal incidence, with a 10 mm gap between the end of the capillary and the targeted surface. On the backside of the BSO was a 260 nm thick conductive ITO layer (circular with diameter of 15 mm) which was connected to a DC power supply to impose a DC bias potential and examine its influence to the plasma–surface interaction. Semi-spherical electrodes on a small spring were used to make the connection with the ITO layer.

A more comprehensive description on the operation of a Mueller polarimeter can be found in literature^25,35^. Both the PSG and PSA consist of a linear polarizer and two externally adjustable retarders (ferro-electric liquid crystals). This allows for the control of the polarization state of the probing light beam. This in turn leads to the capturing of a Mueller matrix of an examined sample. The Mueller matrix gives a mathematical description of the optical properties of the sample following the Stokes’ representation of polarized light instead of Jones’ representation. This means that depolarization is included together with diattenuation and birefringence. The latter was of most importance since it relates directly to the imposed electric field inside the sample due to surface charges as a result of the plasma–surface interaction. Depolarization can be an interesting tool as well since it allows to look at the surface changes a material undergoes during plasma exposure^36^. Example images of the Mueller matrices and logarithmic decomposed matrices can be found in Ref.^25^.

### Calibration and analysis

The system was calibrated before placing the BSO and plasma jet in its center, by measuring and analyzing the Mueller matrix of four calibration samples namely air, two linear polarizers, and a waveplate. This followed the eigenvalue calibration method^37,38^. All the optical properties included in a Mueller matrix are entangled with each other, meaning a decomposition is needed to extract them. The logarithmic decomposition was the most applicable since the polarized light beam was used to examine the sample in transmission and the optical properties were thus obtained as an integral along the optical path through the sample^39,40^.

The logarithmic decomposition separates the total birefringence in a circular component and two linear components, i.e. $\Gamma _{0/90^\circ }$ and $\Gamma _{45/135^\circ }$. These represent the linear birefringence in two different linear polarization states, respectively the horizontal/vertical system and the diagonal system. Both can scale with the external electric field according to the Pockels effect and the orientation of the polarized light beam with respect to the sample^41^. Previously, the following relation between the linear retardances and the electric field components *E* was derived:

1 $$\begin{aligned} \Gamma _{0/90}= & {} \frac{2\pi d^*}{\lambda }\cdot n_o^3\cdot r_{41}\cdot \left( \sin (\theta )E_x - \cos (\theta ) E_z\right) , \nonumber \\ \Gamma _{45/135}= & {} \frac{2\pi d^*}{\lambda }\cdot n_o^3\cdot r_{41}\cdot \sin (\theta )\cdot \cos (\theta )\cdot E_y, \end{aligned}$$

with refractive index $n_o=2.54$, electro-optic constant $r_{41}=4.8$ pm/V, wavelength $\lambda =550$ nm, examination angle $\theta =45$ deg, and optical pathlength $d^*=d/\cos (\theta )$ with thickness $d=0.5$ mm.

In Ref.^26,33^ an experimental procedure was followed to measure the Mueller matrix twice, once with the plasma jet on the front side of the BSO crystal and once on the backside. This allowed for the separation of the individual electric field components from the detected birefringence. This time, however, this was not necessary due to the presence of the ITO layer. This caused the electric field lines to be aligned perpendicular to the surface of the sample. Therefore no indication of the radial electric field components was observed throughout this work and only the axial $E_z$ component was inducing a linear birefringence. Equation (1) can therefore be converted to obtain $E_z$ from the linear retardance. Additionally, the surface charge density $\sigma$ was examined from the electric field profile following

2 $$\begin{aligned} E_z=\frac{\sigma }{\varepsilon _0\varepsilon _r}, \end{aligned}$$

with $\varepsilon _0$ the vacuum electric permittivity and the relative permittivity of BSO $\varepsilon _r=56$. The total charge $\sigma$ is split in the periodical charging $\sigma _p$ occurring each period and continuous charging $\sigma _c$ present due to an applied bias potential. Due to the orientation of the jet and target with respect to the propagation direction of the probing light beam, a positive DC bias will lead to negative continuous surface charge $\sigma _c$ and thus a negative electric field.

### Atmospheric pressure plasma jet

The plasma jet was similar as used in previous works^26,33^. It consisted of a capillary tube (inner diameter = 2.5 mm and outer 4 mm) that housed a stainless steel tube which acted as powered electrode and through which helium flowed. Five millimeters downstream from the end of the stainless steel tube was a ring grounded via a 1 k$\Omega$ resistor (used to measure the current) attached on the outside of the dielectric capillary. This meant the plasma jet was in a coaxial configuration. There was 20 mm left from the grounded ring to the end of the capillary.

The helium flowed at 1 slm and mono-polar positive voltage pulses were used to generate the plasma. The high voltage (HV) pulses were varied in amplitude and pulse width (several $\upmu$s, see Fig. 2b) with a fixed operation frequency of 5 kHz. The high voltage has been detected at the powered electrode and the current was measured over a 1 k$\Omega$ resistor connected to the grounded ring outside of the capillary tube. The current peak at the fall of the slope appears to be greater for the 1.0 $\upmu$s pulse compared to the 5.0 $\upmu$s pulse. This suggests that the surface charges accumulated at the inner side of the capillary decrease during the HV pulse.

In addition to this normal continuous operation mode, in the second part of the paper the plasma jet was also operated in a burst mode where a train of pulses was sent with a frequency of 10 Hz and duty cycle of 70 percent. This entails that the plasma jet was powered by $\upmu$s-monopolar pulses at 5 kHz for 70 ms and then turned off for 30 ms. This was done to examine the dynamics and amount of accumulated surface charge density $\sigma _c$ that is continuously present as a result of the applied DC bias.
